# Supplementary material for: Locoregional Treatments for Metastatic Gastrointestinal Stromal Tumor in British Columbia: A Retrospective Cohort Study from January 2008 to December 2017
Source: Cancers (Basel). 2022 Mar 14;14(6):1477. doi: 10.3390/cancers14061477 (PMC8945875; doi:10.3390/cancers14061477)
Supplement: Supplementary file 1 [file cancers-14-01477-s001.zip › cancers-1620196-supplementary.pdf]

Article

Locoregional Treatments for Metastatic Gastrointestinal Stromal Tumor in British Columbia: A Retrospective Cohort Study from January 2008 to December 2017

Tiffany Patterson [1], Haocheng Li [2], Jocelyn Chai [3], Angeline Debruyns [4], Christine Simmons [5], Jason Hart [6], Phil Pollock [1], Caroline L. Holloway [7], Pauline T. Truong [7] and Xiaolan Feng [3,8,9,*]

Table S1. Systemic treatment of metastatic Gastrointestinal Stromal Tumors (GIST) patients in British Columbia (BC) (Jan 2008 – Dec 2017).

	Treatment Characteristics	Patients that received treatment
Lines of Treatment	Lines of treatment	
	Mean (min – max)	1 (1-6)
	**First line (n=120)**	
	Imatinib	112 (93.3%)
	Sunitinib	6 (5%)
	Regorafenib	1 (0.8%)
	Nilotinib	1 (0.8%)
	**Second Line (n=59)**	
	Sunitinib	52 (88.1%)
	Imatinib	1 (1.7%)
	Regorafenib	6 (10.2%)
	**Third Line (n=33)**	
	Regorafenib	22 (66.7%)
	Imatinib	3 (0.1%)
	Sunitinib	1 (3%)
	Avapritinib	1 (3%)
	Sorafenib	4 (12.1%)
	Nilotinib	1 (3%)
	Clnical trial (imatinib + BKM120)	1 (3%)
	**Fourth Line (n=15)**	
	Imatinib	8 (53.3%)
	Regorafenib	2 (13.3%)
	Sorafenib	1 (6.7%)
	Nilotinib	1 (6.7%)
	Cabozantinib	3 (20%)
	**Fifth Line (n=4)**	
	Imatinib	1 (25%)
	Avapritinib	1 (25%)
	Nilotinib	1 (25%)
	Ripretinib	1 (25%)
	**Sixth Line (n=1)**	
	Imatinib	1 (100%)
